# Parasite Carbohydrate Vaccines

**DOI:** 10.3389/fcimb.2017.00248

**Published:** 2017-06-12

**Authors:** Jonnel A. Jaurigue, Peter H. Seeberger

**Affiliations:** ^1^Department of Biomolecular Systems, Max Planck Institute of Colloids and InterfacesPotsdam, Germany; ^2^Institute for Chemistry and Biochemistry, Freie Universität BerlinBerlin, Germany

**Keywords:** carbohydrate, vaccine, malaria, toxoplasmosis, leishmaniasis

## Abstract

Vaccination is an efficient means of combating infectious disease burden globally. However, routine vaccines for the world's major human parasitic diseases do not yet exist. Vaccines based on carbohydrate antigens are a viable option for parasite vaccine development, given the proven success of carbohydrate vaccines to combat bacterial infections. We will review the key components of carbohydrate vaccines that have remained largely consistent since their inception, and the success of bacterial carbohydrate vaccines. We will then explore the latest developments for both traditional and non-traditional carbohydrate vaccine approaches for three of the world's major protozoan parasitic diseases—malaria, toxoplasmosis, and leishmaniasis. The traditional prophylactic carbohydrate vaccine strategy is being explored for malaria. However, given that parasite disease biology is complex and often arises from host immune responses to parasite antigens, carbohydrate vaccines against deleterious immune responses in host-parasite interactions are also being explored. In particular, the highly abundant glycosylphosphatidylinositol molecules specific for *Plasmodium, Toxoplasma*, and *Leishmania* spp. are considered exploitable antigens for this non-traditional vaccine approach. Discussion will revolve around the application of these protozoan carbohydrate antigens for vaccines currently in preclinical development.

## Introduction

The first vaccine was the smallpox inoculation introduced in 1796 by Jenner that used whole, attenuated organisms to generate a protective immune response. Our ever-increasing understanding of the underlying immune response that drives vaccination has led to modern subunit vaccines that are formulated to exacting standards. These subunit vaccines use defined protein or carbohydrate antigens implicated in virulence and disease to generate a more directed, nuanced immune response against the offending pathogen.

Over two centuries of vaccinology has seen significant progress in protection from many viral and bacterial diseases affecting humans such as polio, *Haemophilus influenza* type B, *Streptococcus pneumoniae*, and *Neisseria meningitis*, saving millions of lives each year. And yet, it is disheartening to consider that no vaccine exists for human parasitic infections that continue to cause suffering in many parts of the world (Nyame et al., 2004; Astronomo and Burton, 2010; Hoffman et al., 2015).

More than a million people die each year from diseases like malaria and leishmaniasis, with lifelong disability, disfigurement, and suffering for those that are living with disease (Hotez et al., 2014). As it stands, diseases caused by protozoan parasites are a leading cause of death the world over yet vaccine strategies for global parasitic diseases such as malaria (John et al., 2008; Seder et al., 2013; Tinto et al., 2015; Gosling and von Seidlein, 2016; WHO, 2016b) and toxoplasmosis (Jongert et al., 2009) have been pursued for decades. The failure in developing an effective vaccine against human parasite infection lies in part to the complexity of parasite biology compared to other microbes (Astronomo and Burton, 2010; Hoffman et al., 2015). Another failure is our relatively poor understanding of the protein and carbohydrate antigens relevant to parasitic virulence. Given that parasite disease pathology often arises from complex host immune responses to parasite antigens, ongoing research to better understand host-parasite interactions in disease is vital to parasite vaccine development (Schofield and Grau, 2005).

Carbohydrates are considered compelling, exploitable targets for vaccination to overcome the challenges that have prevented the realization of a human parasite vaccine (Nyame et al., 2004; Rodrigues et al., 2015). They are abundantly present on the surface of parasites and play a key role in host-parasite interactions (Rodrigues et al., 2015). Unique carbohydrate antigens characterize multiple developmental stages and tend to be immunoreactive for both protozoan and helminth parasites (Nyame et al., 2004). Adding to the appeal of carbohydrate antigens is the ongoing, systematic characterization of parasite glycobiology regarding their structure, function and biosynthesis (Nyame et al., 2004; Rodrigues et al., 2015). In this review, we will discuss the advantages and disadvantages of modern, carbohydrate antigen-based subunit vaccines, and reflect on the latest developments of carbohydrate vaccines for major protozoan parasites.

## Carbohydrate vaccine anatomy

### Carbohydrates antigens

Carbohydrates are abundant on the surfaces of all cells and exist as poly- and oligosaccharides attached to proteins and lipids (Horlacher and Seeberger, 2008). They are involved in key biological processes such as cell adhesion, modulatory processes, and structural functions (Varki and Lowe, 2009). For pathogenic microbes, carbohydrate interactions are utilized for attachment (Kline et al., 2009) and invasion (de Groot et al., 2013). In turn, pathogenic microbial carbohydrates can be recognized by host immune systems to induce the production of carbohydrate-specific antibodies that can serve protective functions (Astronomo and Burton, 2010). Carbohydrates have been exploited for protective vaccination for decades given their crucial roles in development, growth, and disease (Varki and Lowe, 2009).

Carbohydrate biosynthesis is not directly template-driven as is the case for nucleic acid and proteins (Rodrigues et al., 2015). Instead, their biosynthesis is a complex, multi-enzymatic process (Delorenzi et al., 2002) forming linear and branched molecules with varied linkages. The result is a class of biopolymers of great complexity and diversity. Their heterogeneity means that access to pure, defined carbohydrates remains a challenge (Liu et al., 2006; Adibekian et al., 2011; Geissner and Seeberger, 2016). Microbial cell culture can be a readily available biological source of carbohydrate antigens, however carbohydrate isolation can be complex and even tedious (Geissner and Seeberger, 2016). Furthermore, isolation is not so straightforward for carbohydrates of low abundance or when microbial culture is not possible. This is especially true for parasites, which generally require more complex culturing conditions compared to bacteria. Fortunately, carbohydrate synthesis technology continues to advance and is increasingly becoming an alternative to biological isolation to source carbohydrates (Anish et al., 2014). Moreover, carbohydrate synthesis allows for pure, completely defined carbohydrate antigens as a basis for synthetic carbohydrate vaccines.

### Carrier proteins

Virtually all vaccines rely on antibody production and subsequent immunological memory against the target antigen for their protective effect (Zinkernagel, 2003). Accordingly, the immune response against the antigen is a key consideration when it comes to vaccine design where strong, long lasting immune responses are desired. In this regard, proteins and carbohydrates are generally regarded as thymus dependent or thymus independent antigens, respectively, which characterizes the type of antibody immune response they elicit.

#### Thymus dependent and thymus independent antigens

Thymus dependent (TD) antigens, such as proteins, are taken up by antigen presenting cells (APCs). The endocytosed antigen is processed through a series of catalytic steps which liberate small peptide fragments. These peptide fragments form complexes with MHCII molecules and thereby are able to be displayed on the MHCII molecules of the APC. T cells specific to the displayed MHCII-peptide complex are co-stimulated by the APC (Avci and Kasper, 2010). The activated T cells go on to “help” the antigen-specific B cells, promoting their proliferation, affinity maturation, antibody isotype switching and long lasting immunological memory (Pulendran and Ahmed, 2011).

Carbohydrate antigens are classed as thymus independent (TI) antigens and are poorly immunogenic compared to TD antigens (Weintraub, 2003). Carbohydrate antigens are recognized by APCs through pathogen recognition receptors (Blander and Sander, 2012), endocytosed, and processed into oligosaccharide epitopes. These oligosaccharides are not presented on MHCII molecules, but instead are presented directly on the APC cell surface to activate carbohydrate antigen-specific B cells. Due to the lack of T cell activation, B cells are activated in the absence of affinity maturation and isotype switching (Mond et al., 1995) which leads to the predominant production of low affinity IgM antibodies, and only low levels of IgG (Mond and Kokai-Kun, 2008; Astronomo and Burton, 2010; Berti and Adamo, 2013). The IgM antibodies bind in the micromolar range, compared to TD antigen-derived IgG antibodies that bind in the nanomolar range (Broecker et al., 2016; Geissner et al., 2016). B and T cell memory is often not achieved and the immune response is short lived (Adams et al., 2008; Mond and Kokai-Kun, 2008; Hütter and Lepenies, 2015). Moreover, children less than 2 years of age fail to mount an antibody immune response to TI antigens (Weintraub, 2003; Landers et al., 2005).

#### Glycoconjugates

In the 1920s and 1930s, covalent conjugation of carbohydrate antigens to protein scaffolds was first explored to investigate the interaction between the TD antigen properties of proteins with TI carbohydrate antigens (Avery, 1931). This early work formed the basis of the first glycoconjugate carbohydrate vaccines produced in the 1980s (Schneerson et al., 1980; Beuvery et al., 1982; Wessels et al., 1993), where bacterial capsular polysaccharides (CPS) were covalently linked to so-called carrier proteins. Immunization with these CPS-protein glycoconjugates enabled T cell-mediated B cell activation against the target carbohydrate antigen, and induced long term immune memory even in infants (Stein, 1992).

**Figure 1:**
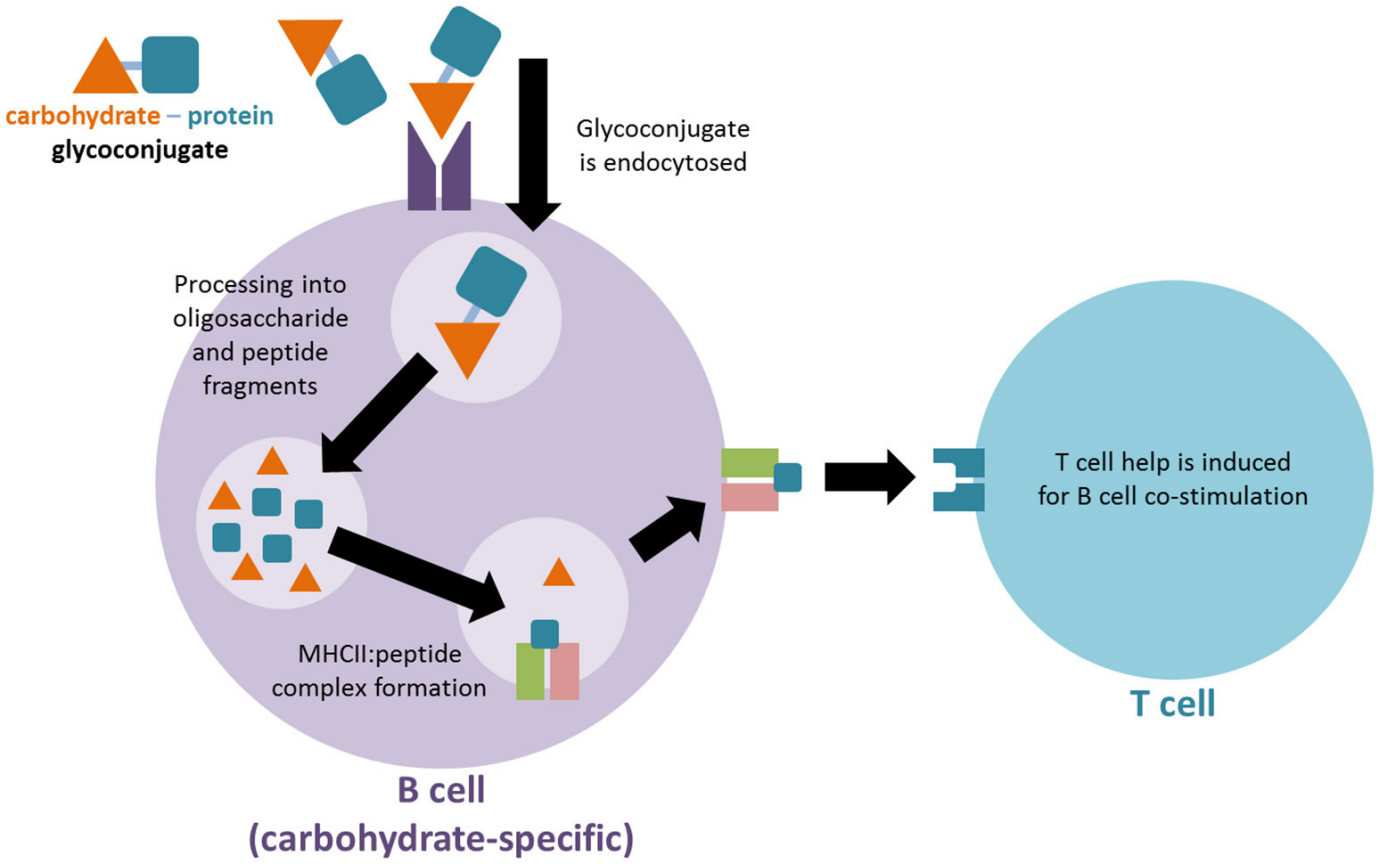
Proposed mechanism for immune activation of T cells by glycoconjugate vaccines. The carbohydrate-protein conjugate is recognized by carbohydrate-specific B cells. The protein component of the conjugate is subsequently endocytosed, processed and presented on MHCII molecules of the carbohydrate specific B cell in order to activate a T cell for co-stimulation.

The mechanism whereby TI carbohydrate antigens can activate the immune system like TD protein antigens via glycoconjugation remains an open area of research. A long-standing mechanistic explanation argues that the carbohydrate-protein conjugate is recognized by carbohydrate-specific B cells. The protein component of the conjugate is subsequently endocytosed, processed and presented on MHCII molecules of the carbohydrate specific B cell. Through the MHCII-peptide complex a peptide-specific T cell can activate the carbohydrate-specific B cell, resulting in TD immune responses against the desired carbohydrate antigen (Figure 1; Lucas et al., 2005). A more recently proposed second mechanism argues that after uptake by carbohydrate-specific B cells the glycoconjugate is processed into glycopeptide fragments. Thus, the peptide portion of the fragment can form an MHCII-peptide complex, enabling the simultaneous presentation of the hydrophilic carbohydrate portion to carbohydrate-specific T cells. The T cell interaction activates the presenting B cell accordingly (Avci et al., 2011).

Five TD carrier proteins are currently used in licensed carbohydrate vaccines (Pichichero, 2013). Of these licensed carrier proteins, a non-toxic mutant of diphtheria toxin (CRM_197_) is often used in vaccine development research for parasitic diseases. Another carrier protein often used in parasite vaccine development is keyhole limpet haemocyanin (KLH). Unlike many other carrier proteins KLH is itself highly glycosylated and is a good promotor of TD and TI responses (Nyame et al., 2004), however it is not licensed for human use.

The linker conjugating the carrier protein to the carbohydrate antigen is another key consideration of carbohydrate vaccines. Synthetic carbohydrates with defined carrier protein binding sites are advantageous for generating well-defined glycoconjugates with predictable conjugation sites. To minimize the immunogenicity of the linker (Buskas et al., 2004; Gotze et al., 2015) short linkers with no functional groups are best used to ensure that immune responses against the desired antigen epitope are not detrimentally affected. The processing of the linker after uptake by APCs is also an important consideration, to allow for the release of vaccine oligosaccharide and peptide moieties for antigenic display.

### Adjuvants

Adjuvants are substances that modulate or bolster an effective immune response against the antigens in the vaccine. Formulations are typically emulsions and vesicles that can serve as a delivery vehicle for antigen vaccine components and allow for the slow release of vaccine antigen components over time. Adjuvants can assist antigen immunogenicity by increasing local inflammation and antigen uptake by APCs, and aid in their migration to lymph nodes (Petrovsky and Aguilar, 2004; Di Pasquale et al., 2015). Ideally, adjuvants reduce the amount of antigen or number of immunizations needed for vaccination (Petrovsky and Aguilar, 2004).

Aluminum-based mineral (Alum) salts served as the only adjuvants for human vaccines for decades (Zimmermann and Lepenies, 2015) and are used for a wide range of vaccines that aim to predominantly induce antibody-mediated immune responses (Bhowmick et al., 2014). Despite its worldwide use, research on its mechanism of action is ongoing (De Gregorio et al., 2008). Today, alum, virosomes, lipid A derived adjuvants, and squalene adjuvants are all in use for human licensed vaccines (Astronomo and Burton, 2010; Di Pasquale et al., 2015; Zimmermann and Lepenies, 2015) and are commonly used in vaccine research. Crude Freund's adjuvant (CFA) is a powerful immunogen often used as an adjuvant for candidate parasite vaccines, but it is not approved for licensed human vaccines.

Modern vaccines that use better defined, or even synthetic antigens, are generally less immunogenic than crude, whole organism vaccines. Research is ongoing in advancing the efficacy of adjuvant systems for vaccines (Petrovsky and Aguilar, 2004). Many of these new adjuvant systems that are currently being tested in bacterial vaccines are of great interest for adaption to candidate vaccines of parasite diseases. For example, an adjuvant used in animal models, α-GalCer, was covalently attached to *S. pneumoniae* CPS to act as both carrier and adjuvant for the carbohydrate antigen (Cavallari et al., 2014). Endosomal processing by carbohydrate-specific B cells displays α-GalCer antigens via CD1d, stimulating iNKT cells (a class of T cells) to promote B cell hypermutation, class-switching and immunological memory (Cavallari and De Libero, 2017). Zwitterionic polysaccharides (ZPSs) are another emerging class of potentially self-adjuvanting carrier isolated from commensal anaerobic bacteria. ZPSs have the special property of containing one positive and one negative charge on adjacent monosaccharides. When they are processed by APCs this crucially enables their presentation on MHCII complexes, leading to T cell activation. Thus, they are able to activate adaptive immune responses for conjugated carbohydrate antigens in the absence of carrier protein (Berti and Adamo, 2013), leading to the possibility of fully-carbohydrate vaccines (Nishat and Andreana, 2016).

### The success of carbohydrate vaccines

The concept of carbohydrate antigen vaccines started in the 1920s with the first published evidence that “residue antigens” of *Streptococcus pneumoniae* were the CPS of the bacterium (Heidelberger and Avery, 1923, 1924), later demonstrated to be important for virulence and serotype specificity (Hütter and Lepenies, 2015). The CPS of *S. pneumoniae* was shown to produce CPS-specific antibodies (Tillett and Francis, 1929) that protected against the disease symptoms of *S. pneumoniae* (Hütter and Lepenies, 2015), leading to the first CPS antigen vaccine against *S. pneumoniae* in 1947 (Grabenstein and Klugman, 2012). The advent of antibiotics at around the same time somewhat stalled vaccine research, as antibiotics became the preferred method for bacterial disease prevention (Grabenstein and Klugman, 2012; Hütter and Lepenies, 2015). The rise of antibiotic resistance in the following decades (Davies and Davies, 2010) led to a resurgence in carbohydrate vaccine research (Vliegenthart, 2006).

In the 1970s and 80s, CPS-based vaccines for *S. pneumoniae* were licensed and approved in the USA and Europe. Increasing numbers of strain-specific CPS were added to further itinerations of the vaccine to increase its efficacy, culminating into a 23-valent CPS antigen vaccine first licensed in 1983, protecting vaccinated adults against 87% of *S. pneumoniae* disease in the USA (Grabenstein and Klugman, 2012; Cavallari and De Libero, 2017). Glyconjugate carbohydrate vaccines were later introduced from the 1990s onward to allow for vaccination of broader demographics, especially infants. Glycoconjugate carbohydrate vaccines against disease caused by *H. influenzae* type b infection lead to its virtual elimination within countries with widespread coverage (Lindberg, 1999). Similarly, the use of glycoconjugate vaccines against Neisseria meningitides has also met with effective results (Girard et al., 2006). Vaccines for *S. pneumoniae* are also now glycoconjugates. Several other glycoconjugate vaccines are currently under development, such as a carbohydrate vaccine for group B streptococcus (De Gregorio and Rappuoli, 2014; Lepenies, 2015). Today, glycoconjugate vaccines have substituted pure carbohydrate vaccines where possible, with the most common carrier proteins being CRM_197_ and tetanus toxin (Cavallari and De Libero, 2017).

## Exploiting carbohydrate antigens for protozoan parasite vaccines

The key components of CPS carbohydrate vaccines—antigen, carrier protein, linker, and adjuvant—have remained largely consistent since their inception. Current parasite vaccine research follows the same component model in this regard. However, as illustrated in the following sections, a recurring theme for carbohydrate parasite vaccines is the need to steer away from traditional prophylactic vaccine development strategies that were successfully applied to bacterial infections.

In addition to carbohydrate vaccines that can induce sterile protection against the parasite itself, vaccinating against deleterious immune responses in host-parasite interactions is another strategy. Much of the pathology of parasitic disease is due to the host's own immune responses against the parasite. For example, the deadly manifestation of severe malaria is a result of the host's own immune response against parasite-derived molecules, causing the toxic, hyperinflammatory response associated with severe malaria (Boutlis et al., 2002; Krishnegowda et al., 2005; Patel et al., 2007). The *Plasmodium falciparum*-specific glycoform of glycosylphosphatidylinositol (GPI) was identified as the putative toxin implicated in disease (Schofield and Hackett, 1993). GPI molecules are present on the surface of virtually all eukaryotic cells and serve as surface protein anchors, but parasite-specific GPIs occur at relatively high levels in parasitic protozoa (Gowda, 2002). Vaccination strategies aimed at neutralizing the effect of this malaria toxin are being pursued (Schofield, 2007). Similar carbohydrate vaccine strategies for other protozoan parasites are also employed (Buxbaum, 2013). Anti-toxin vaccines have proven successful for other microbial infections, such as the diphtheria toxoid vaccine (Playfair et al., 1990; Schofield, 2007).

We will explore the latest developments for both traditional and non-traditional carbohydrate vaccine approaches for three of the world's major protozoan parasitic diseases—malaria, toxoplasmosis, and leishmaniasis.

### Plasmodium

Malaria is an intra-erythrocytic parasitic disease caused by *Plasmodium* protozoan species. It is among the most devastating infectious diseases of human history and over 3.3 billion people across 97 countries are at risk of infection today (WHO, 2013b, 2014). An estimated 198 million new clinical cases of malaria occur globally with over 80% of new clinical cases occur in sub-saharan Africa alone (WHO, 2013b). Severe malaria disease develops in 5% of *P. falciparum* infections and accounts for 98% of all malaria-related deaths. There are an estimated 584,000 deaths per year attributed to severe disease, mostly young children under the age of 5 (WHO, 2013b). Estimates of severe disease-related deaths are as high as 1.2 million (Murray et al., 2012). Without a vaccine, at least €2.4 billion are spent annually on malaria control programs using bed nets, insecticides and drug treatments (WHO, 2013b).

A malaria vaccine should be possible considering that naturally acquired immunity to disease symptoms develops over time. Vaccines against malaria aim to reduce morbidity and mortality, and should be effective in protecting against severe malaria. In the long term, the vaccine should also protect against all clinical disease (WHO, 2013a). To this end, many stages of the parasite lifecycle are targeted by vaccines that decrease parasite load. Examples of some prophylactic vaccines include the whole *P. falciparum* sporozoite vaccine currently in field trials (Seder et al., 2013), and the protein antigen based RTS,S vaccine which is the most advanced example of malaria vaccine currently under development in Phase III (John et al., 2008; Tinto et al., 2015; Gosling and von Seidlein, 2016) and Phase IV trials (WHO, 2016b). At present, the RTS,S vaccine has not been licensed for use as a malaria vaccine (WHO, 2016a).

#### Anti-toxin vaccine

Severe malaria pathology is largely considered to arise from toxic effects of *P. falciparum* GPI (PfGPI), which exists either as protein free glycolipids, or as the major carbohydrate modifications for proteins essential for erythrocyte invasion (Gowda et al., 1997; Schofield and Grau, 2005). PfGPI-specific antibodies are found in adults of malaria-endemic areas, and may be inhibiting the ability of PfGPI to induce the hyper-inflammatory response associated with severe malaria. This is still up for debate, since studies that find an association between PfGPI-specific antibody titer and protection from severe malaria (Brasseur et al., 1990; Naik et al., 2000; Gowda, 2002; Keenihan et al., 2003; Perraut et al., 2005) are balanced by studies that find no such association (de Souza et al., 2002; Boutlis et al., 2005; Cissoko et al., 2006; Gomes et al., 2013; Mbengue et al., 2016). However, PfGPI-specific antibodies are reported to show relevant action in modulating immune responses by protecting immune cells against severe *P. falciparum-*induced inflammatory responses *in-vitro* (Schofield et al., 1993; de Souza et al., 2010).

**Figure 2:**
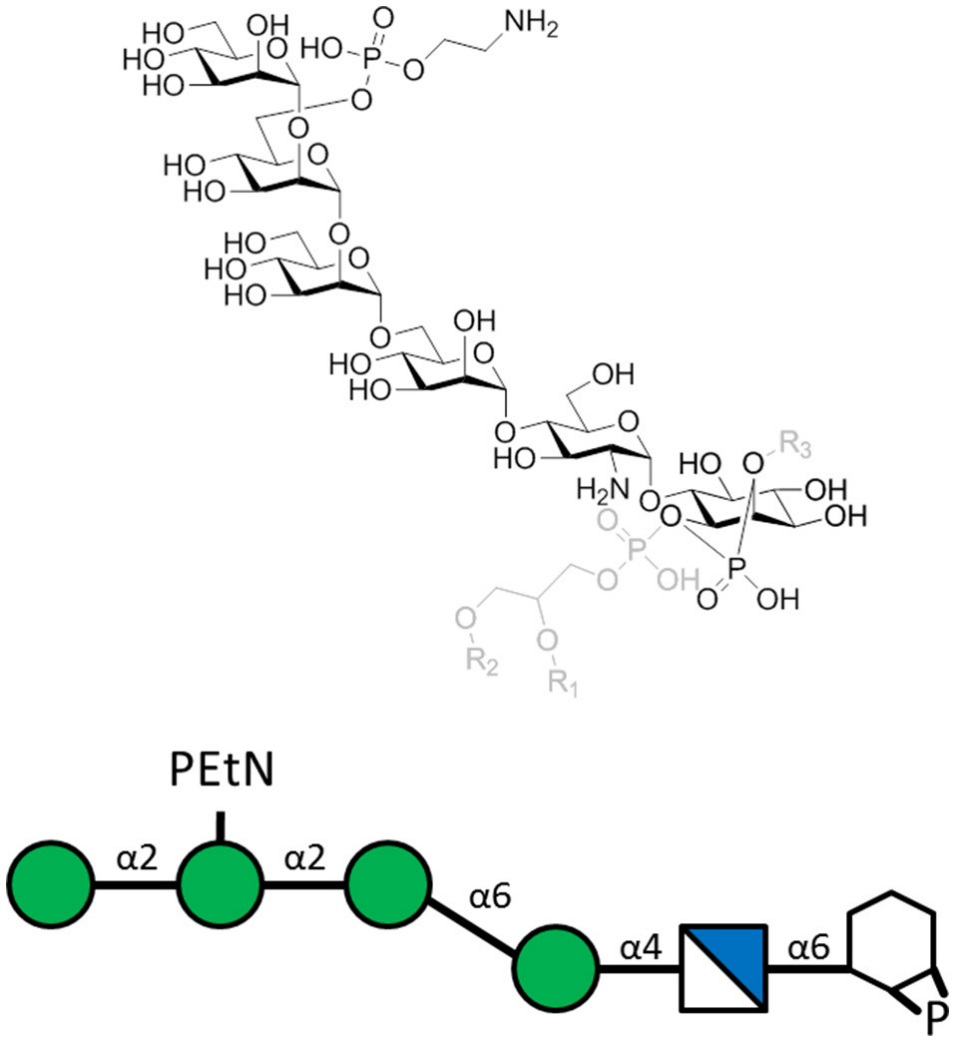
The *P. falciparum* GPI hexasaccharide of the sequence α-Man-(1-2)-α-[PEtN-6]Man-(1-2)-α-Man(1-6)-α-Man-(1-4)-α-GlcN-(1-6)-*myo*-Ino-1,2-cyclic-phosphate that was chemically synthesized, conjugated to KLH, and used for immunization. The carrier protein was attached to the top PEtN moiety, where PEtN is an abbreviation for phosphoethanolamine. The full PfGPI (in gray) is shown for context, where R groups denote lipid moieties.

To evaluate the effect of using PfGPI in an anti-toxin vaccine, a PfGPI hexasaccharide was synthesized, conjugated to KLH carrier protein and emulsified in CFA (Figure 2). C57BL/6 mice were immunized with the glycoconjugate, and then challenged with *P. berghei* ANKA in a mouse model of malaria. Immunization resulted in significant protection against severe malaria, with clearly reduced death rates at 75% survival. The immunized mice were also protected from acidosis, pulmonary oedema, cerebral syndrome and fatality characteristic of the disease model. Apparently, the induction of protective, PfGPI-specific antibodies ameliorated a hyper-inflammatory response against the PfGPI toxin, specifically shown to neutralize the parasite-induced production of TNF-α by macrophages *in vitro*. Furthermore, vaccination did not change parasitaemia levels, demonstrating that the effect of the carbohydrate vaccine candidate was through the neutralization of GPI's toxic effects, rather than interfering with parasite replication (Schofield et al., 2002).

Following this work, synthetic PfGPI were utilized as biomarkers to improve our understanding of the PfGPI-specific antibody response (Kamena et al., 2008; Tamborrini et al., 2010). The synthesis of PfGPI molecules continues to be explored (Gurale et al., 2016).

#### Sterile immunity vaccine

**Figure 3:**
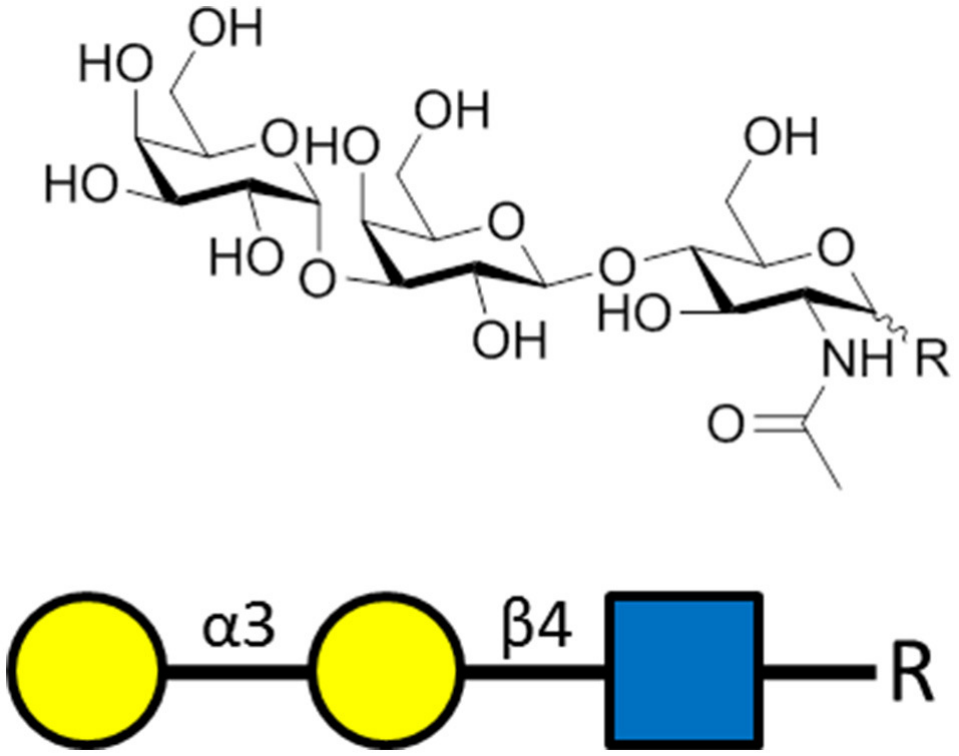
The α-gal carbohydrate epitope of the sequence α-Gal-(1-3)-β-Gal-(1-4)-GlcNAc-R that was used as the antigen for generating α-gal-specific antibodies.

In healthy adults, up to 1–5% of circulating IgG and IgM are specific to the carbohydrate known as α-Gal (Figure 3; Macher and Galili, 2008). In contrast to other mammals, humans do not express α-Gal (Galili and Swanson, 1991) allowing for immune reactivity to this carbohydrate (Galili et al., 1984). Exposure to microbiota expressing this glycan drives production of α-Gal-specific antibodies (Macher and Galili, 2008) which are believed to contribute to immune resistance against α-Gal-expressing pathogenic microbes (Bishop and Gagneux, 2007; Cywes-Bentley et al., 2013). *P. falciparum* and other animal model *Plasmodium* spp. express the α-Gal carbohydrate (Galili et al., 1998) possibly bound to GPI-anchored surface proteins (Yilmaz et al., 2014). Early studies already uncovered an association between α-Gal-specific IgM and protection from *P. falciparum* infection in humans. Thus, the effect of immunization on the production of α-Gal-specific antibodies was investigated (Yilmaz et al., 2014).

Genetically modified mice unable to express α-Gal (Yang et al., 1998) were immunized for the production of α-Gal IgG and IgM. The immunizations were either inoculation with α-Gal expressing *E. coli* bacteria, α-Gal rich rabbit RBCs, or synthetic α-Gal conjugated to BSA. Adjuvants used were CFA and toll-like receptor 9 agonists that enhanced the immunogenicity. The mice were then challenged with *P. berghei* ANKA infection by *Anopheles* mosquito bites. It was found that α-Gal immunization reduced the risk of parasite transmission, thereby providing sterile immunity. This sterile immunity appears to be due to the cytotoxic action of α-Gal specific IgM and subclasses of IgG against the inoculating parasites. Moreover, the antibodies were shown to inhibit hepatocyte transmigration of the parasite (Yilmaz et al., 2014).

Sterile immunity against malaria infection through the induction of α-Gal-specific antibodies is not similarly present for people living in malaria endemic regions, possibly due to low levels of naturally acquired, protective α-gal-specific antibodies. Promoting a T cell-dependent immune response against the α-Gal carbohydrate was shown to enhance the protective effect of these antibodies in the mouse model (Yilmaz et al., 2014). Moreover, naturally acquired α-gal-specific antibodies may enhance the immunogenicity of antigens enriched with α-gal epitopes by augmenting the T cell response following immunization. This suggests that coupling the α-Gal carbohydrate to existing malaria vaccine candidates could enhance their immunogenicity (Benatuil et al., 2005; Yilmaz et al., 2014). The effect of α-Gal carbohydrate vaccination against other protozoan parasites expressing α-Gal, such as *Trypanosoma* spp. and *Leishmania* spp., may also be considered (Yilmaz et al., 2014).

### Toxoplasma

*Toxoplasma gondii* is found worldwide, capable of infecting nucleated cells of many warm-blooded animals (McLeod et al., 2009; Debierre-Grockiego and Schwarz, 2010) and is estimated to infect half of the world's population (Liu et al., 2012). The disease burden for humans has been well-documented (McLeod et al., 2009). Transmission to humans is either through consumption of food contaminated with tissue cysts and meat products from infected animals or by ingestion of oocysts released in the feces of infected cats (Kijlstra and Jongert, 2008).

Immunocompetant individuals tolerate *T. gondii* infection which is either asymptomatic or manifest with mild flu-like symptoms. However, the formation of parasite-containing tissue cysts prevents the clearance of the parasite after infection (Montoya and Liesenfeld, 2004). When latent carriers of *T. gondii* are later immunocompromized they are at risk of severe inflammatory reactions in the brain and central nervous system after liberation of the parasites from these cysts (Luft and Remington, 1992). Pregnant women encountering their first infection can also transmit the parasite to the unborn child leading to retardation or abortion (Remington et al., 2004).

Early vaccination strategies using live parasites (Cutchins and Warren, 1956) and fixed parasites (Krahenbuhl et al., 1972) protected from subsequent challenge. Today, vaccines against toxoplasmosis aim to limit acute parasitemia, protect against congenital toxoplasmosis, reduce the number of tissue cysts, or lessen parasite transmission (Kur et al., 2009). To date, sterile immunity against *T. gondii* has not been achieved (Jongert et al., 2009). A live vaccine for veterinary toxoplasmosis exists to give limited protection during pregnancy (Buxton and Innes, 1995) but is not approved for human use and does not fully eliminate the parasite (Liu et al., 2012).

#### GPI vaccine

Glycolipid GPI anchors of *T. gondii* (TgGPI) have been considered as possible vaccine antigens due to their effect in modulating inflammatory TNF-α responses against the parasite (Debierre-Grockiego, 2010). In 2015, TgGPI were explored as possible vaccine candidates for the first time.

**Figure 4:**
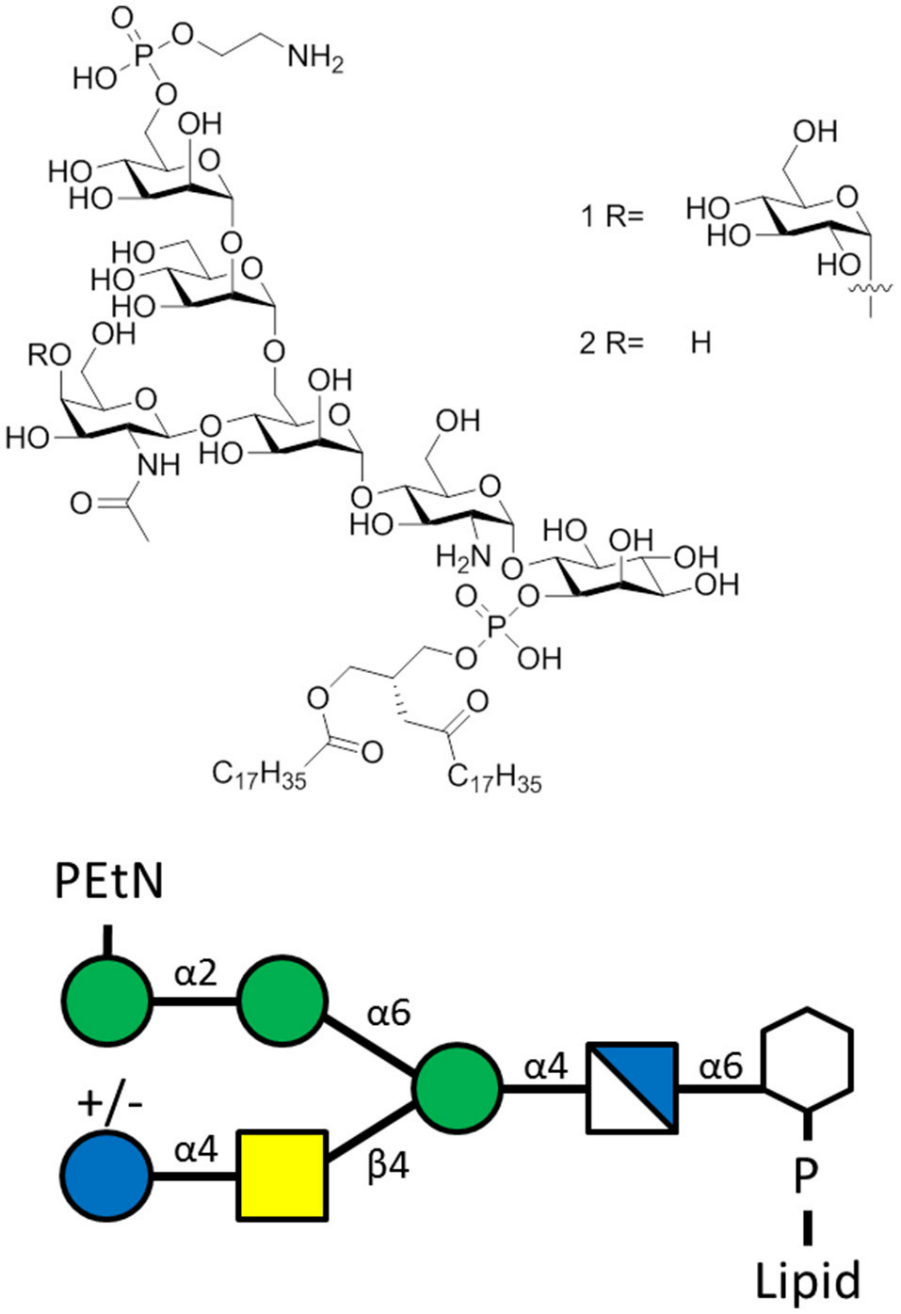
The *T. gondii* GPI of the sequence α-Man-(1-2)-α-Man-(1-6)-α-Man-(1-4)-α-GlcN-(1-6)-*myo*-Ino with lipid moiety. Glycoform 1R bears a α-Glc(1-4)-β-GalNAc side chain, while glycoform 2R lacks the glucose moiety.

GPI anchors are highly abundant on *T. gondii* (~10^6^ copies per cell) (Tsai et al., 2012) and exist as a protein-attached or protein-free glycoform (Figure 4). Both glycoforms induce inflammatory reactions like macrophage TNF-α production (Debierre-Grockiego et al., 2003) through TLR-2 and TLR-4 signaling (Debierre-Grockiego et al., 2007), which exacerbates toxoplasmosis in mice (Hunter et al., 1996). A vaccine that induces TgGPI-specific antibodies could ameliorate TgGPI-mediated inflammatory effects and lower disease burden (Debierre-Grockiego, 2010).

The two major TgGPI glycoforms were synthethized and covalently conjugated to CRM_197_. BALB/c mice were then immunized with either one of the glycoconjugates before challenge with virulent *T. gondii* RH strain. Immunization did not provide protection for mice in the lethal challenge model. Furthermore, the induced antibodies failed to exhibit any significant effect on the inflammatory response for either group (Gotze et al., 2015). Analysis of the immune response indicates that antibody induction was directed away from the desired carbohydrate side branch of the TgGPI, and more toward the linker used to attach the carbohydrate to the carrier protein. In a potential next step, the formulation has to be adjusted (Gotze et al., 2015).

Ongoing research utilizing synthetic TgGPI molecules has helped to identify biomarkers of acute and latent infection. During acute infection, high levels of TgGPI-specific IgM and IgG are present, while latent infection shows a reduced IgM response (Gotze et al., 2014).

### Leishmania

There are two million new cases of leishmaniasis every year as the disease is increasingly becoming a worldwide health burden (Desjeux, 2004). The vector borne, facultative intracellular parasite (Chappuis et al., 2007) enters mononuclear, phagocytotic cells such as macrophages. Cutaneous leishmaniasis is the most common form of disease, notable for skin ulcers that result in disability and scarring for the infected patient (Seeberger, 2007; WHO, 2017). *Leishmania donovani* causes visceral leishmaniasis and is the most severe form of disease characterized by fever, substantial weight loss, anemia, swelling of liver and spleen, and possible death. It is believed to be second only to malaria in terms of fatal infection (Seeberger, 2007; Aebischer, 2014). Patients are treated with antimony drugs which are costly, toxic and increasingly ineffective against resistant parasite strains. A vaccine against leishmaniasis is a desirable, economic strategy to combat this disease (Lee et al., 2012; Singh et al., 2016).

A cocktail of heat-killed *Leishmania* parasites is a clinically tested vaccine. However, the efficacy of the vaccine to prevent disease is not confirmed (Armijos et al., 2004; Velez et al., 2005). Other attempts involving killed or attenuated parasites for leishmanial vaccine development have not resulted in a licensed vaccine (Topuzogullari et al., 2013).

#### Vaccine efforts

**Figure 5:**
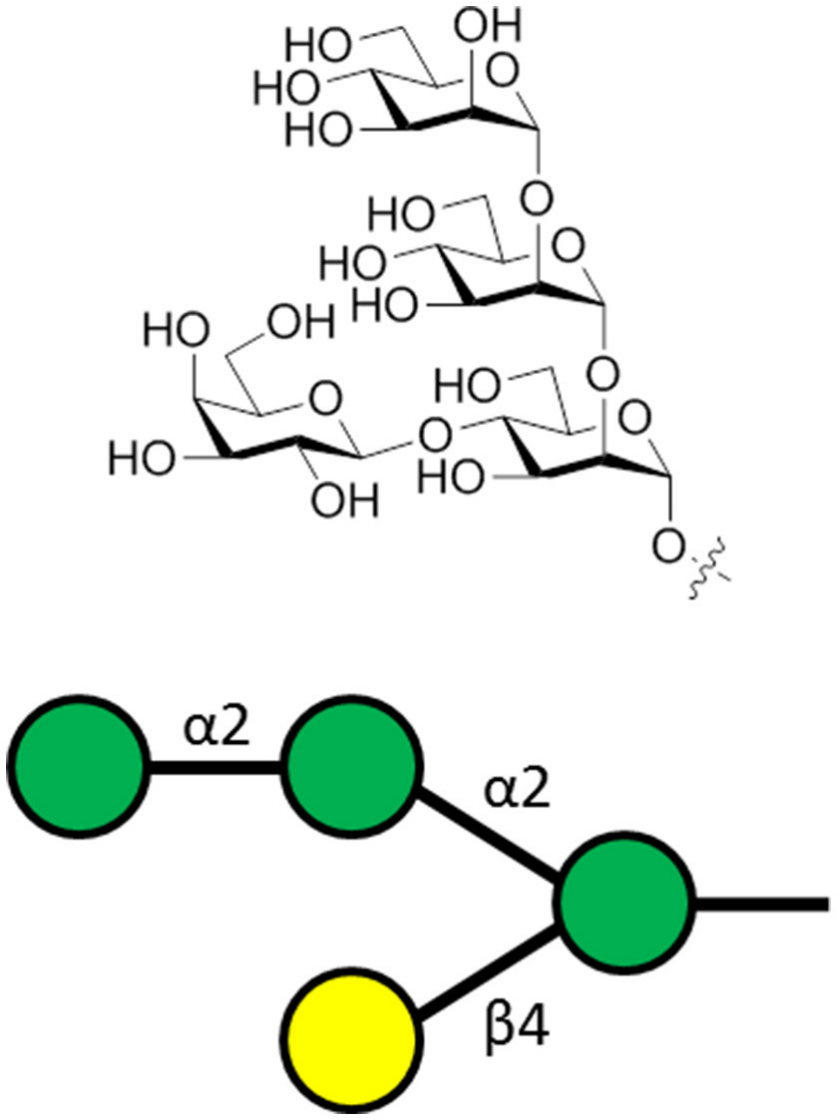
The general structure of the capping tetrasaccharide α-Man-(1-2)-α-Man-(1-2)-[β-Gal-(1-4)]-α-Man of *Leishmania* LPG. This oligosaccharide is attached to phosphoglycan repeating units, followed by a GPI anchor.

*Leishmania* parasites express lipophosphoglycans (LPG) on their cell surface. This molecule is composed of a GPI anchor, a repeating phosphorylated disaccharide fragment, and variable cap oligosaccharides (Seeberger, 2007). The LPGs are important for survival and virulence of the parasite (Spath et al., 2000) and vaccination preparations with purified LPG is protective against cutaneous leishmaniasis (McConville et al., 1987; Russell and Alexander, 1988; Moll et al., 1989). Regarding the variable capping oligosaccharide, a unique capping tetrasaccharide (Figure 5) was identified as vital for parasite invasion of macrophages (Descoteaux and Turco, 2002) and was the key component of a synthetic carbohydrate vaccine for leishmaniasis (Liu et al., 2006).

The capping oligosaccharide moiety of LPG was synthesized in initial immunological studies exploiting this antigen for vaccination. The carbohydrate was loaded onto virosomes that served as an integrated carrier and adjuvant, before being used to immunize BALB/c mice. Oligosaccharide-specific IgM and IgG1 responses were produced, indicating that the immune response to the carbohydrate antigen was T cell-dependent. Furthermore, the antibodies against the synthetic carbohydrate were cross-reactive with natural carbohydrate antigens of *Leishmania* parasites indicating its possible utility as a vaccine antigen (Liu et al., 2006). A further study immunized BALB/c mice with synthetic LPG capping oligosaccharides conjugated to CRM_197_ and emulsified in CFA. This vaccine candidate produced IgG antibodies specific for the parasite. The oligosaccharides were used to evaluate immune responses of infected humans and dogs as the basis for a diagnostic test (Anish et al., 2013). Given the carbohydrate vaccines currently in production, animal model challenge studies to test the protective effect of these synthetic molecules is the next logical step.

LPG vaccination to protect against cutaneous leishmaniasis employed animal challenge studies that evaluated the LPG component of whole *L. amazonensis* antigen (LaAg). It was known that intramuscular, systemic immunization of LaAg results in deleterious disease outcomes after challenge, however LPG depletion rendered LaAg protective against *Leishmania* infection with respect to lesion growth and parasite load compared to non-depleted LaAg. This indicated that LPG was the component responsible for deleterious effects of LaAg inoculation. During further investigations, mice were intranasally vaccinated with LPG alone to determine whether intranasal vaccination of the disease-promoting component could promote protection against cutaneous leishmaniasis (Pinheiro et al., 2007). Intranasal vaccination with LPG was protective, and provided further evidence for the potential of utilizing LPG in a carbohydrate antigen vaccine that would protect against cutaneous leishmaniasis.

**Figure 6:**
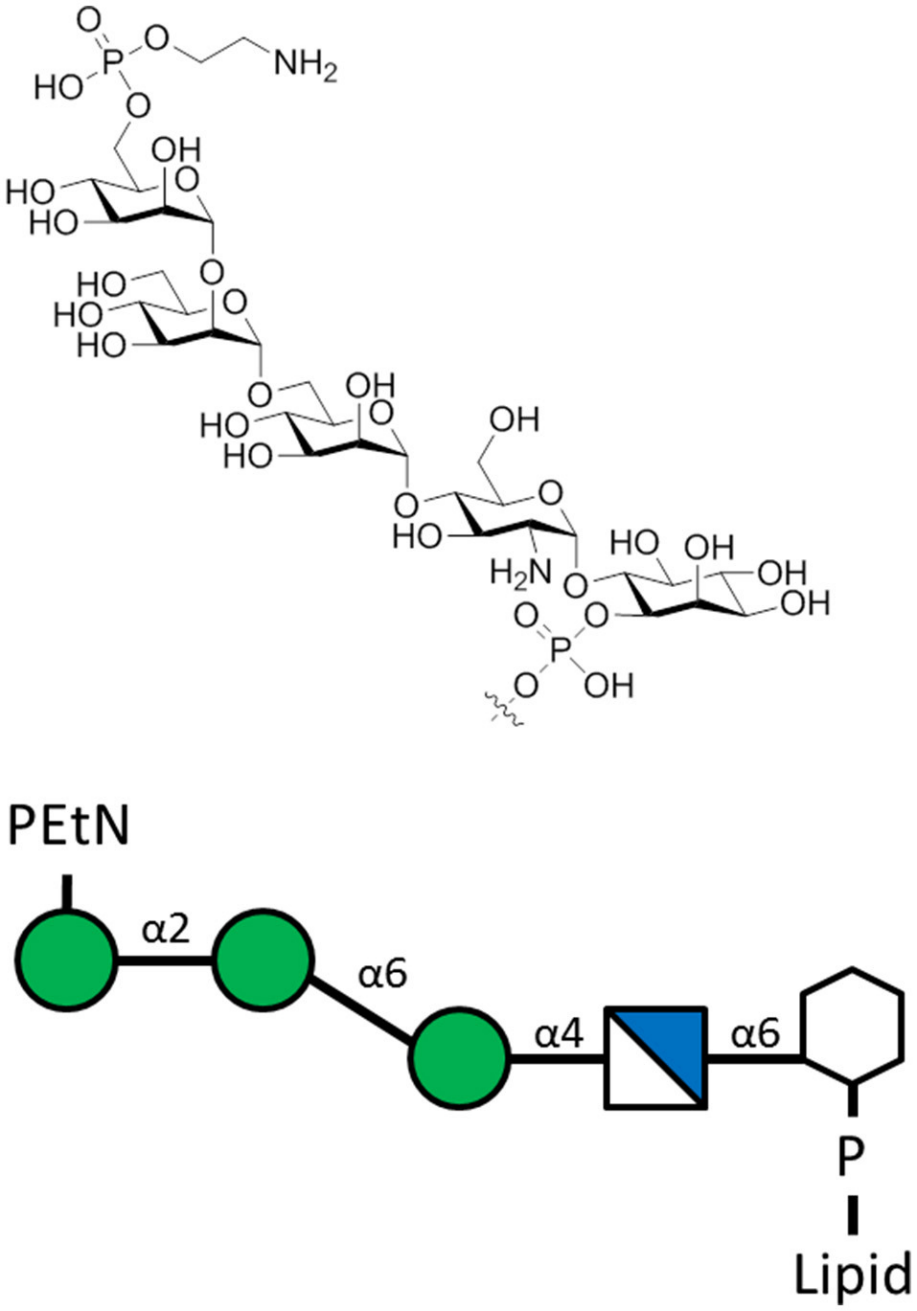
The *L. mexicana* GIPLs contain the sequence PEtN-6-α-Man-(1-2)-α-Man-(1-6)-α-Man-(1-4)-α-GlcN-(1-6)-*myo*-Ino-2-phosphate attached to a lipid base that anchors into the membrane (McConville et al., 1993). PEtN is an abbreviation for phosphoethanolamine. Additions to this base sequence are found for many of the GIPLs. Note the structure of the carbohydrate chain is the same as the sequence of protein-free *P. falciparum* GPI anchors (Assis et al., 2012).

Recently, the role of *L. mexicana* protein-free GPI molecules (GIPL) in disease has been elucidated, shedding more light on immunologic pathways affecting glycolipid-specific antibody responses (Figure 6). After it was determined that *L. mexicana* infection induces GIPL-specific IgG1 responses, a monoclonal antibody against GIPL was shown to bind to the surface of parasites, and promote IL-10 production in macrophages co-cultured with parasite. Production of IL-10 is deleterious as it blocks an effective immune response that is needed to kill parasites and resolve skin lesions in cutaneous leishmaniasis. In humans, GIPL-specific antibodies are produced in response to infection with cutaneous leishmaniasis. Opsonization of parasites with these antibodies was shown to promote deleterious IL-10 production in macrophages. The role of GIPL-specific antibodies in both mouse and humans opens the possibility of generating a carbohydrate vaccine that can induce competing, non-pathogenic antibody isotypes that can protect against *L. mexicana* infection (Buxbaum, 2013).

## Concluding remarks

Carbohydrate vaccines represent a promising application of glycobiology to human health. The immense successes of bacterial CPS vaccines should, in theory, be similarly achievable with parasite carbohydrate vaccines. With continued advances in parasite culture handling, and carbohydrate synthesis technologies, it is a great time to apply the development strategies employed for bacterial CPS vaccine research to parasite carbohydrate vaccine research.

## Author contributions

JJ contributed to the drafting of the article. PS and JJ contributed to all revisions of the article.

### Conflict of interest statement

The authors declare that the research was conducted in the absence of any commercial or financial relationships that could be construed as a potential conflict of interest.
